# Combined in vitro/in vivo genome-wide CRISPR screens in triple negative breast cancer identify cancer stemness regulators in paclitaxel resistance

**DOI:** 10.1038/s41389-023-00497-9

**Published:** 2023-11-06

**Authors:** Gang Yan, Meiou Dai, Sophie Poulet, Ni Wang, Julien Boudreault, Girija Daliah, Suhad Ali, Jean-Jacques Lebrun

**Affiliations:** Department of Medicine, Cancer Research Program, McGill University Health Center, Montreal, QC H4A 3J1 Canada

**Keywords:** Breast cancer, Chemotherapy

## Abstract

Triple negative breast cancer (TNBC) is defined as lacking the expressions of estrogen receptor (ER), progesterone receptor (PR), and human epidermal growth factor receptor 2 (HER2). TNBC patients exhibit relatively poor clinical outcomes due to lack of molecular markers for targeted therapies. As such chemotherapy often remains the only systemic treatment option for these patients. While chemotherapy can initially help shrink TNBC tumor size, patients eventually develop resistance to drug, leading to tumor recurrence. We report a combined in vitro/in vivo genome-wide CRISPR synthetic lethality screening approach in a relevant TNBC cell line model to identify several targets responsible for the chemotherapy drug, paclitaxel resistance. Computational analysis integrating in vitro and in vivo data identified a set of genes, for which specific loss-of-function deletion enhanced paclitaxel resistance in TNBC. We found that several of these genes (ATP8B3, FOXR2, FRG2, HIST1H4A) act as cancer stemness negative regulators. Finally, using in vivo orthotopic transplantation TNBC models we showed that FRG2 gene deletion reduced paclitaxel efficacy and promoted tumor metastasis, while increasing FRG2 expression by means of CRISPR activation efficiently sensitized TNBC tumors to paclitaxel treatment and inhibited their metastatic abilities. In summary, the combined in vitro/in vivo genome-wide CRISPR screening approach proved effective as a tool to identify novel regulators of paclitaxel resistance/sensitivity and highlight the FRG2 gene as a potential therapeutical target overcoming paclitaxel resistance in TNBC.

## Introduction

Triple negative breast cancer (TNBC) has the worst clinical prognosis of all breast cancer molecular subtypes. These tumors do not express hormone receptors or human epidermal growth factor receptor-2 (HER-2). They account for around 15–20% of all breast cancer and do not respond to targeted therapies such as endocrine therapy. As such, chemotherapy, which can be administered first-line or in the adjuvant and neoadjuvant settings, often remains the only option for TNBC patients [1, 2]. Taxols (paclitaxel and docetaxel) are microtubule-stabilizing agents which exert strong anti-tumor effects through blocking activation of the spindle checkpoint, also called mitotic checkpoint, further leading to mitotic arrest and apoptosis without cell division [3]. Taxols are used for clinical treatments for ovarian, breast, lung, cervical, and pancreatic cancer patients. In particular and in the context of breast cancer, paclitaxel is often used first-line for the treatment of TNBC patients [4].

While chemotherapy (i.e., paclitaxel) remains the main resort for TNBC, patients often fail to respond to sustained treatments and eventually develop resistance to the drug. Previous studies in various tumor types indicated that chemoresistance could arise from both pre-existing clonal cancer cell populations and from acquired mutations [5–8]. As a result, despite showing strong initial anti-tumor effects, paclitaxel efficacy is often limited or reduced due to resistance mechanisms [9]. This represents a major limitation of the use and efficacy of chemotherapy in TNBC patients. As such, it is critical to define the molecular mechanisms and target genes underlying paclitaxel resistance in breast cancer, particularly TNBC. A recent study showed that TNBC chemoresistance is likely determined by pre-existing selective advantages in various subclones although transcriptional reprogramming takes place in response to chemotherapy [10]. In particular, induction of the ATP-dependent efflux pump P-glycoprotein (ABC1 or MDR1) was found to mediate chemoresistance in ovarian and breast cancer [11, 12]. Cancer stem cells (CSCs) or tumor-initiating cells represent a unique subpopulation of cancer cells that have the capacity to self-renew. CSCs are highly resistant to drug treatments and also contribute to chemoresistance by overexpressing P-glycoprotein efflux pump [13]. Other examples of transcriptional reprogramming leading to chemoresistance involve activation of the oncogene, EGFR [14], deletion of the tumor suppressor, TP53 [15], and promotion of epithelial-mesenchymal transition (EMT) [16]. Thus, to overcome paclitaxel resistance and improve TNBC patient clinical outcomes, it is vital to identify those genes and mechanisms providing TNBC cells with selective advantages toward paclitaxel treatment.

CRISPR-Cas9 technology has come to rise as a new gene editing tool that can efficiently generate loss-of-function mutations by introducing double strand breaks (DSBs) at the genomic level. As such, the use of unbiased, forward genetic in vivo CRISPR screening approaches, at the genome-wide scale has proven to be a powerful tool to identify cancer vulnerabilities [17–19]. In this study we performed genetic loss-of-function in vitro and in vivo CRISPR screens in TNBC, at the genome-wide scale, using paclitaxel as a positive selection pressure. Bioinformatics and data analysis cross-referencing in vitro and in vivo genome-wide screen datasets uncovered 34 common candidate genes in the positive selection. We further showed that CRISPR-induced specific loss-of-function deletion of these genes led to paclitaxel resistance in TNBC cells. Interestingly, we found several of these genes (ATP8B3, FOXR2, FRG2, HIST1H4A) to act as cancer stemness regulators, able to regulate cancer stem cell self-renewal activity and expression of the endothelial protein C receptor (EPCR), a specific stemness marker for TNBC [20, 21]. We also showed that FRG2 gene deletion reduced paclitaxel efficacy and promoted tumor metastasis in an in vivo orthotopic transplantation TNBC model. Moreover, we found, FRG2 over-expression through specific activation of the endogenous FRG2 gene promoter, using CRISPR/dCas9 Synergistic Activation Mediator (SAM) system, efficiently sensitized TNBC tumors to paclitaxel treatments and inhibited their metastatic abilities, further highlighting the FRG2 gene as a potential therapeutic target to overcome paclitaxel resistance in TNBC.

## Results

### In vitro and in vivo genome-wide pooled sgRNA library screens in triple negative breast cancer

To start identifying novel potential genes contributing to resistance against paclitaxel, we performed pooled genome-wide CRISPR/Cas 9 loss-of-function screens both in vitro and in vivo using highly tumorigenic SUM159PT (hereafter referred to as SUM159) TNBC cells. SUM159 is a mesenchymal TNBC cell line carrying both TP53 and PI3KCA mutations, the two most frequently mutated genes in TNBC patients [17, 22, 23], with an estimated prevalence of 74% and 17% respectively [17, 24]. Moreover, most TNBC patients with PIK3CA mutation also carry TP53 mutation, accounting for 12% of all TNBC patients [17]. Interestingly, these patients harboring both mutations also exhibit the worst overall survival outcomes [17]. As such, the SUM159 cell line adequately reflects the most aggressive genetic features of TNBCs. These further highlight the representation power of the SUM159 cell line as a TNBC model. We previously used this TNBC model system to identify new cancer vulnerabilities and a novel potential targeted therapy for TNBC [17].

**Fig. 1:**
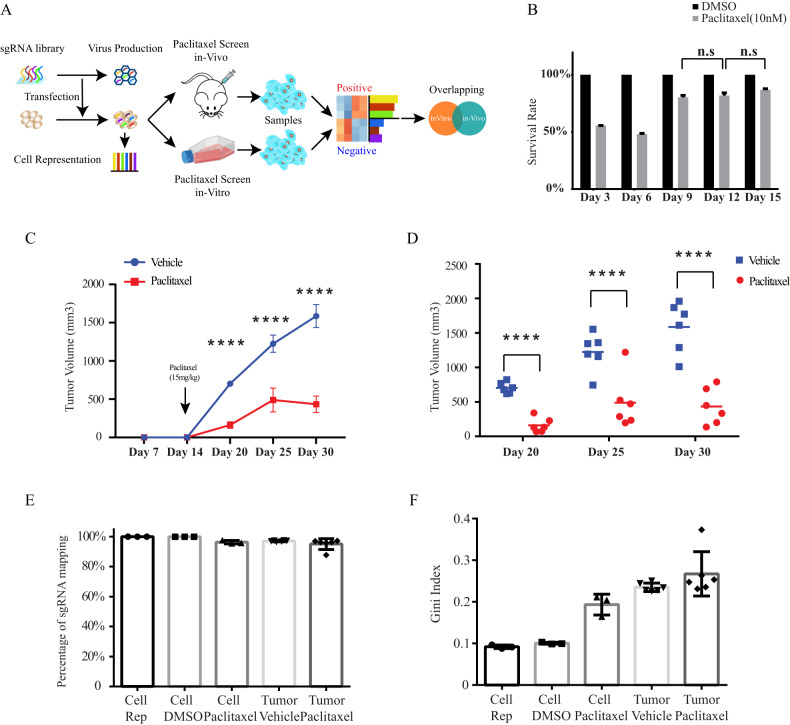
In vitro and in vivo genome-wide pooled sgRNA library screens in triple negative breast cancer. **A** Graphical overview of the genome-wide CRISPR/Cas9 loss-of-function screen performed in in vivo and in vitro. **B** The in vitro cell survival rates of the library infected cells after paclitaxel treatment every 3 days in three independent experiments. Survival rate was calculated by normalizing paclitaxel-treated to DMSO-treated cells. Data are presented as mean ± SD and Student’s *t* test is used to determine the *p* value between the survival rates of Day 9, Day 12, and Day 15 (*n* = 3). **C**, **D** 30 million library infected SUM159 cells were subcutaneously transplanted into each NSG mouse followed by weekly treatment of paclitaxel (15 mg/kg) or vehicle for 3 weeks. **C** Tumor growth curve of NSG mice treated with vehicle or paclitaxel in three independent experiments and data are presented as mean ± SEM (*n* = 6, 2 replicates for each experiment). **D** The individual tumor volume at each timepoint (*n* = 6). Student’s *t* test is used to determine the *p* value. **E**, **F** Quality measurements of the cell and tumor sequencing samples. **E** The sgRNA-mapping percentage of the cell (*n* = 3) and tumor (*n* = 6) sequencing samples at the endpoint. **F** The Gini index of the cell and tumor sequencing samples. Data are presented as mean ± SD. n.s. *p* > 0.05, *p < 0.05, **p < 0.01, ***p < 0.001, or ****p < 0.0001.

Both CRISPR screens were performed at the genome-wide level, using the GeCKOv2 lentiviral library (detailed information is included in the “Methods”), as previously shown [17]. For each screen (in vitro/in vivo), three independent experiments were performed. Briefly, as illustrated in Fig. 1A, SUM159 cells were subjected to spin-infection at a multiplicity of infection (MOI) of ~0.3. Infected cells were selected in the presence of puromycin of 2 µg/ml for 7 days. Samples were collected after puromycin selection for cell representation, while the rest of the cells were used for the in vitro and in vivo screens.

### In vitro screen

Forty million cells were treated with 10 nM paclitaxel or vehicle (DMSO) as a selection pressure and maintained in cell culture for another 2 weeks. As shown in Fig. 1B, cell viability was assessed every 3 days and cell survival rate was calculated by normalizing paclitaxel-treated to DMSO-treated cells. Nine days following the start of paclitaxel treatment cells exhibited resistance to the drug. Drug treatment was extended for another week, to ensure the stability of paclitaxel resistance before cells were collected.

### In vivo screen

Thirty million cells infected with the GeCKOv2 lentiviral library were transplanted subcutaneously in NOD SCID Gamma (NSG) immunodeficient mouse. Once tumor became palpable (2 weeks following transplantation) mice were separated into two groups (6 mice per group) and the drug selection pressure was applied with either paclitaxel (15 mg/kg; intraperitoneal injection) or vehicle alone, once per week for 3 weeks. Tumor growth and volumes were monitored at regular intervals (Fig. 1C). Paclitaxel treatment efficiently and significantly reduced tumor growth to reach a plateau 25 day post-transplantation, presumably resulting from acquired resistance mechanisms. Drug injections continued for another week to ensure that drug treatment did not further reduce tumor volumes. At experimental endpoint (30 days), tumors were excised and collected. At all-time points tumor size was significantly reduced in paclitaxel injected animals compared to controls (Fig. 1D).

### Sample processing

Following collection of cell and tumor samples, genomic DNA was extracted from all samples including the cell representation group and prepared for next generation sequencing (NGS). The quality of the screens was assessed and quantified by mapping sequencing data to the GECKO V2 library (cell/tumor samples vs. library representation). Sequencing data analysis revealed a sgRNA library mapping rate at over 99% with a Gini index below 0.1 for the cell representation samples, indicative of sufficient library presence and of an equal sgRNA distribution before the start of drug selection (Fig. 1E, F). These data indicated that all sgRNAs are well represented, ensuring that specific dynamic changes observed for individual sgRNA are the result of the drug selection pressure rather than the lack of representation during tumor development. As expected, paclitaxel-treated samples (cell paclitaxel and tumor paclitaxel) exhibited higher Gini index compared to vehicle treated samples (Cell DMSO/Tumor Vehicle) reflecting a statistical dispersion of the library distribution, following enrichment or depletion of specific sgRNAs, under paclitaxel selection pressure (Fig. 1F).

### Overlapping in vitro and in vivo datasets identifies 34 candidate genes as paclitaxel sensitizers

**Fig. 2:**
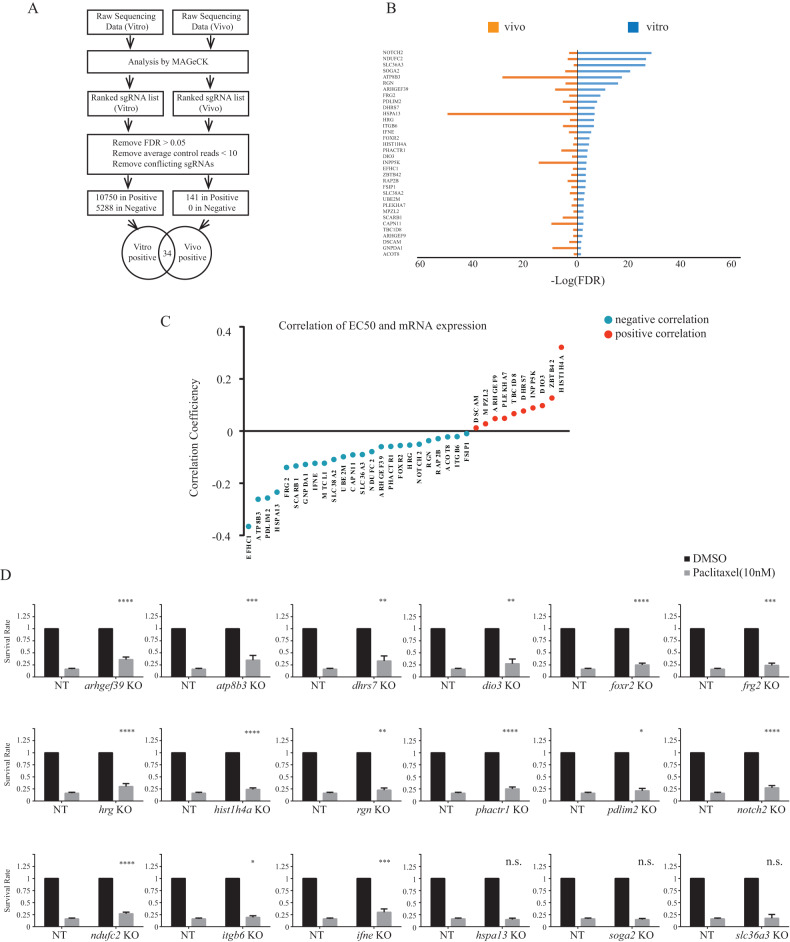
Overlapping in vitro and in vivo datasets identifies 34 candidate genes as paclitaxel sensitizers. **A** The outline of data analysis by integrating in vivo and in vitro sequencing data. **B** The −Log10(FDR) of the common candidate genes identified by overlapping the in vivo (left) and in vitro (right) positive selection. The data was ranked by in vitro data significance level (FDR). **C** The distribution of the correlations between mRNA expression and paclitaxel response (EC50) of 34 genes. mRNA expression of the 34 genes is obtained from CCLE mRNA data and paclitaxel EC50 is from PRISM projects. Blue color indicates negative correlation while red color indicates positive (detailed in Methodology). **D** The cell viability assay to evaluate cell survival rate of the 18 candidate individual knockouts and non-targeting (NT) control with or without paclitaxel treatment (10 nM). Cell survival rate of each single KO was calculated by normalizing paclitaxel-treated cells to DMSO-treated. Student’s *t* test is used to determine the significance level (*p* value) between each KO’s survival rate and NT’s. n.s. *p* > 0.05, *p < 0.05, **p < 0.01, ***p < 0.001, or ****p < 0.0001.

In vivo and in vitro screen were analyzed separately using MAGeCK (Model-based Analysis of Genome-wide CRISPR-Cas9 Knockout) and sgRNAs were ranked according to false discovery rate (FDR) values [25]. Cut-off criteria for selection of potential sgRNA candidates included (1) FDR <0.05; (2) control average read counts above 10 and (3) removal of conflicting sgRNAs (sgRNAs targeting one specific gene but appearing in opposite rank lists (positive or negative). The positive selection identified enriched sgRNAs in cell (in vitro) and tumor (in vivo) samples (10,750 and 141 sgRNAs, respectively). These sgRNAs prevented paclitaxel from working efficiently, thereby defining the genes they target as potential drug sensitizers. The negative selection identified dropout sgRNAs corresponding to genes potentially inducing resistance to paclitaxel (Fig. 2A). sgRNA lists and quantitative analysis tables for negative (in vitro) and positive (in vitro/in vivo) selection are provided in Supplementary Files 1–3). No significant dropouts were found in the in vivo screen. Thus, further analysis specifically focused on potential paclitaxel sensitizing genes from the positive selection. To then shortlist our top candidates the in vivo and in vitro datasets were cross-referenced and overlapped, leading to the identification of 34 common target genes (Fig. 2A, B).

Because these candidate genes represent potential drug sensitizers, we postulated that their respective expression levels should reflect TNBC cells’ response to paclitaxel. To address this, we integrated two public datasets from CCLE (Cancer Cell Line Encyclopedia) and PRISM (Profiling Relative Inhibition Simultaneously in Mixtures) projects and investigated the linear relationship between mRNA expression and paclitaxel response in breast cancer cell lines [26, 27]. As shown in Fig. 2C, for most genes (24 of 34), low target mRNA expression negatively correlated with paclitaxel EC50, suggesting that decreased expression of these genes likely caused paclitaxel resistance, further highlighting them as potential paclitaxel sensitizers.

To experimentally validate this, and as a proof-of-concept, we individually knocked-out (CRISPR/Cas9) the top ranking 18 genes and assessed the paclitaxel (10 nM) response in SUM159 TNBC cells, using a PrestoBlue fluorescence cell viability assay. A non-targeting (NT) gRNA KO was used as negative control. As shown in Fig. 2D, most specific individual KOs (15 out of 18) treated with paclitaxel showed a significant increase in cell survival rate compared to NT sgRNA, confirming these gene KOs contributed to paclitaxel resistance.

### Several candidate genes are involved in cancer stemness

**Fig. 3:**
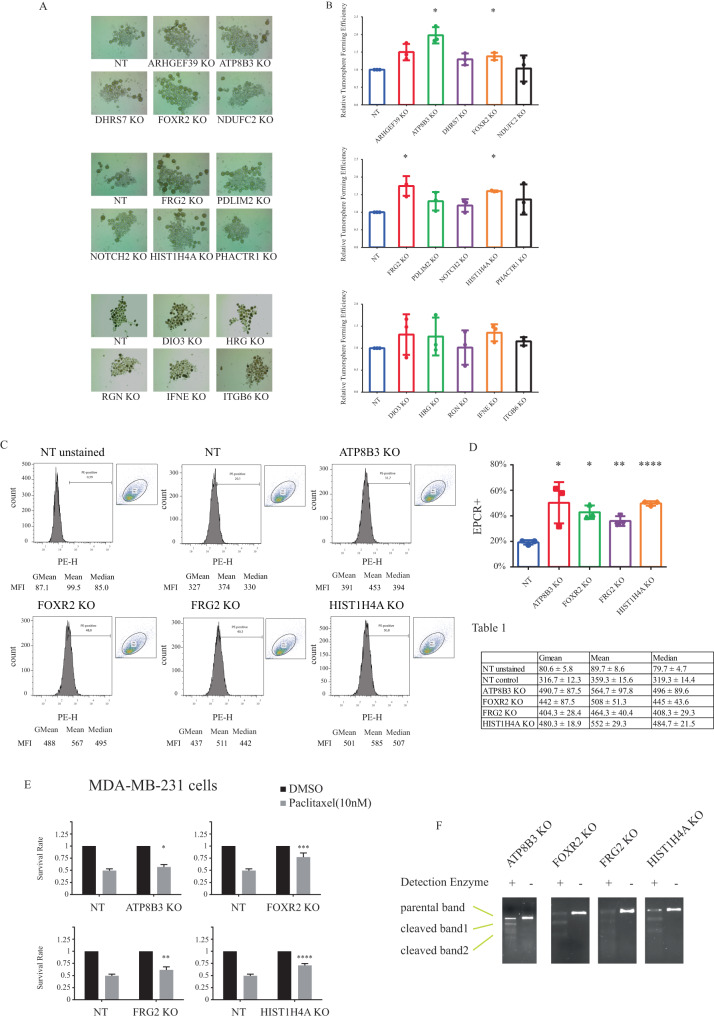
Several candidate genes are involved in cancer stemness. **A**, **B** SUM159 cells were infected with NT or the KO lentivirus individually targeting 15 candidate genes. Tumorsphere assay was performed in the presence of 20 ng/ml EGF, 20 ng/ml bFGF, and B27 for 7 days. Tumorsphere forming efficiency was calculated as number of spheres divided by number of cells seeded. Tumorsphere forming efficiency was further normalized to NT cells. **A** Representative images of tumorsphere assay. **B** Quantification of tumorsphere assay. **C**, **D** Flow cytometry of SUM159 cells of ATP8B3 KO, FOXR2 KO, FRG2 KO, HIST1H4A KO, and NT. An anti-EPCR conjugated to PE was used in flow cytometry assay. Percentage of the EPCR positive (EPCR+) subpopulation was graphed (**C**) and quantified (**D**) using FlowJo. The mean fluorescence intensity (MFI) parameters (GMean: Geometric mean, Mean, Median) of the PE-H channel for each knockout condition and respective mean ± SD values were calculated from three independent flow cytometry experiments and are listed in Table 1. **E** The cell viability assay to evaluate MDA-MB-231 cell survival rate of the four selected individual knockouts (ATP8B3 KO, FOXR2 KO, FRG2 KO and HIST1H4A KO) and non-targeting (NT) control with or without paclitaxel treatment (10 nM). Cell survival rate of each single KO was calculated by normalizing paclitaxel-treated cells to DMSO-treated. Student’s *t* test is used to determine the significance level (*p* value) between each KO’s survival rate and NT’s. **F** Genomic modification of SUM159 cells with individual knockout targeting ATP8B3, FOXR2, FRG2 and HIST1H4A were examined using surveyor (cleavage) nuclease assay. All experiments are performed in three independent times (*n* = 3). The data are presented as mean ± SD and Student’s *t* test is used to determine the *p* value (*n* = 3). n.s. *p* > 0.05, *p < 0.05, **p < 0.01, ***p < 0.001, or ****p < 0.0001.

Breast tumors are heterogenous and contain a unique and rare subpopulation of cancer cells that have the ability to self-renew and exhibit tumor-initiating capacity. This breast cancer stem cell (BCSC) subpopulation features the expression of stem cell markers such as CD24^low^/CD44^High^, aldehyde dehydrogenases (ALDH) [2, 28] and largely contributes to tumor propagation, drug resistance and tumor relapse [29]. Previous studies have emphasized the essential role played by cancer stem cells in chemotherapy resistance [30–32] and the use of chemotherapy on breast cancer cells was found to lead to an enrichment in breast cancer stem cells [33]. Interestingly, we also previously found that BCSC enrichment in TNBC can lead to paclitaxel resistance and that targeting BCSCs could overcome chemoresistance and sensitize TNBC cells to chemotherapy [34]. To thus investigate whether our identified candidate target genes were involved in regulating breast cancer stemness, we assessed the capacity of their individual KOs to regulate BCSC self-renewal ability, using tumorsphere assay in SUM159 cells. The tumorsphere assay is a standard in vitro method to measure and quantify the tumor-initiating capacity of cancer cells, cultured in a growth factor-defined medium under low attachment conditions [35]. Interestingly, as shown in Fig. 3A, B, quantitative analysis revealed that gene silencing of 4 of the 15 genes (ATP8B3, FOXR2, FRG2 and HIST1H4A) significantly increased tumorsphere forming efficiency compared to non-targeting sgRNA KO, highlighting these genes as potential breast cancer stemness regulators. We further analyzed the effects of these 4 genes on stemness, by assessing their effects on endothelial protein C receptor (EPCR). EPCR also known as activated protein C receptor (APC receptor) is a protein encoded by the *PROCR* gene in humans [36, 37]. EPCR is a transmembrane receptor involved in the anticoagulation process that can trigger anti-inflammatory and anti-apoptotic responses [38]. EPCR was identified as a marker of multipotent mouse mammary stem cells (MaSCs). EPCR^+^ cells exhibit a mesenchymal phenotype and enhanced colony-forming abilities [39]. In the breast cancer context, EPCR^+^ TNBC cells exhibit stem cell-like properties and show enhanced tumor-initiating activity [40]. EPCR is highly expressed in aggressive basal-like breast cancer and used as a specific marker for CSCs in TNBC [20, 21]. Interestingly, all individual ATP8B3, FOXR2, FRG2 and HIST1H4A KOs significantly increased EPCR positive (EPCR+) cell numbers (Fig. 3C, D). The mean fluorescence intensity (MFI) values and corresponding statistical analyses for each PE-H channel are indicated for the three parameters (Gmean: Geometric Mean, Mean, Median) and were calculated based on three independent experiments (Table 1). These results are also consistent with our tumorsphere assay data (Fig. 3A, B) as well as with previous studies linking enhanced breast cancer stemness to paclitaxel treatment failure [2, 41, 42]. To further broaden the scope of our results and avoid the limitation of the use of a single cell line, we then examined the effects of knocking out our 4 selected candidates (ATP8B3, FOXR2, FRG2 and HIST1H4A) on the paclitaxel response in another TNBC cell line, MDA-MB-231 using cell viability assays. As shown in Fig. 3E, while paclitaxel efficiently inhibited cell viability in the control non-targeting (NT) infected cells, this response was significantly antagonized when either of the 4 candidate genes were knocked out, using CRIPSR gene editing. These results are consistent with our data obtained in the SUM159 TNBC cell line (Fig. 2D), also showing significant antagonistic effects of the paclitaxel response when those genes were silenced, using CRISPR gene editing. Finally, to ensure that proper indel mutations were inserted into the genomic DNA of our validated CRISPR-KOs, we performed DNA cleavage Surveyor assays. As shown in Fig. 3F, indel mutations were properly inserted for all 4 KO constructs. Collectively, and combined with our findings, showing increased paclitaxel resistance in these gene KOs (Fig. 2), our results define ATP8B3, FOXR2, FRG2 and HIST1H4A as cancer stemness negative regulators, consistent with a role for these genes as potential drug (paclitaxel) sensitizers (Fig. 2).

### Candidate gene KOs block paclitaxel response and increase metastasis in vivo

**Fig. 4:**
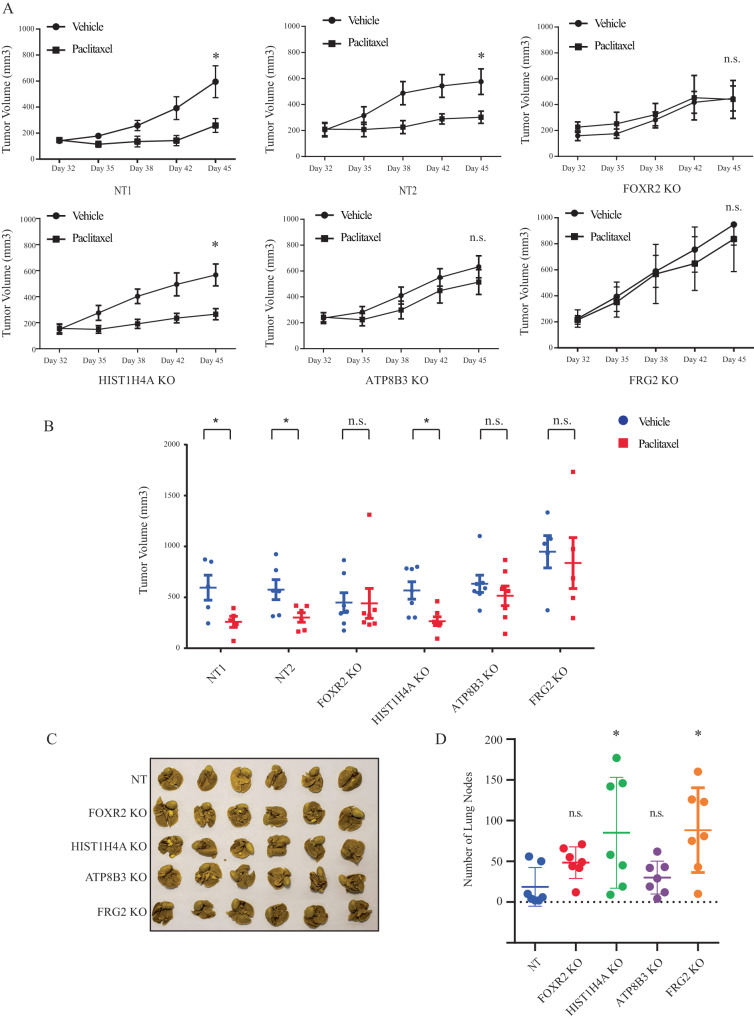
Candidate gene KOs block paclitaxel response and increase metastasis in vivo. **A**, **B** The in vivo orthotopic model of breast cancer to assess the FOXR2, ATP8B3, HIST1H4A, FRG2 individual KO and NT (NT1 and NT2) cells’ response to paclitaxel treatment in NSG mice. Within each KO group, mice were divided into vehicle and paclitaxel treatment arms (5–7 mice for each arm) with similar average tumor volume. The mice were subjected to vehicle or paclitaxel treatment (10 mg/kg) twice per week. Tumor growth curve (**A**) at different timepoints is represented as mean ± SEM. Individual tumor volume (**B**) at experiment endpoint. The *p* values are calculated by the two-sided Student’s *t* test. n.s. *p* > 0.05, *p < 0.05. **C**, **D** The individual KO cells of FOXR2, ATP8B3, HIST1H4A, FRG2 and NT were intravenously transplanted via tail vein injection. The image (**C**) and quantification (**D**) of NT, FOXR2 KO, ATP8B3 KO, HIST1H4A KO and FRG2 KO lung metastatic nodules. Data are presented as individual dot plots and mean ± SD (*n* = 6). The *p* value is calculated by the Mann–Whitney *U* test. n.s. *p* > 0.05, *p < 0.05.

Having shown that ATP8B3, FOXR2, FRG2 and HIST1H4A KOs increased paclitaxel resistance and cancer stemness in vitro, we next investigated whether these KOs could also regulate paclitaxel effects in vivo. For this, SUM159 FOXR2, HIST1H4A, ATP8B3 and FRG2 KO cells were orthotopically transplanted in the mammary fat pad (MFP) of immunodeficient NSG mice, as previously described [17]. Non-targeting (NT) gRNAs were used as negative controls. After 3 weeks, when tumor became palpable, mice were treated with paclitaxel (10 mg/kg) or vehicle alone, twice a week. Interestingly, as shown in Fig. 4A, while paclitaxel treatment led to significant decrease in tumor volume in all control animals (NT1, NT2), the ATP8B3, FOXR2, and FRG2 knockouts showed a complete reversal of the paclitaxel treatment effects. Only HIST1H4A knockout did not show significant reversal effects, although it did show a trend in this direction. Figure 4B representing individual tumor size distribution across all animals in the different groups at experimental endpoint show results consistent with our in vitro data. These results indicate that FOXR2, ATP8B3, and FRG2 gene silencing significantly blocks response to paclitaxel treatment in vitro as well as in preclinical in vivo models of TNBC xenografts.

From a clinical perspective, drug resistance is the leading cause of treatment failure and subsequent distant metastasis occurrence. Because drug resistance leads to enhanced migratory capacity of tumor cells and increased metastatic rates [43, 44], and because cancer stem cells are a main cause for cancer metastasis, we next investigated whether FOXR2, HIST1H4A, ATP8B3, FRG2 KOs could also modulate the metastatic process and lung colonization. For this, NSG mice were inoculated intravenously (tail vein) with NT or FOXR2, HIST1H4A, ATP8B3, FRG2 KOs SUM159 cells. Four weeks following injection, animals were sacrificed, and lungs were resected to assess metastasis, by counting metastatic nodules post-Bouin solution fixation, as we previously showed [17, 45]. As shown in Fig. 4C, D, by study endpoint, both HIST1H4A and FRG2 gene silencing significantly (Mann–Whitney *U* test) increased lung metastatic nodule counts, while the FOXR2 and ATP8B3 KOs both also showed a trend toward increased lung nodules, but not reaching significance. These results highlight the FRG2 and HIST1H4A genes as potent metastatic regulators in TNBC.

### Endogenous activation of FRG2 gene expression sensitizes tumor to paclitaxel and inhibits metastasis

**Fig. 5:**
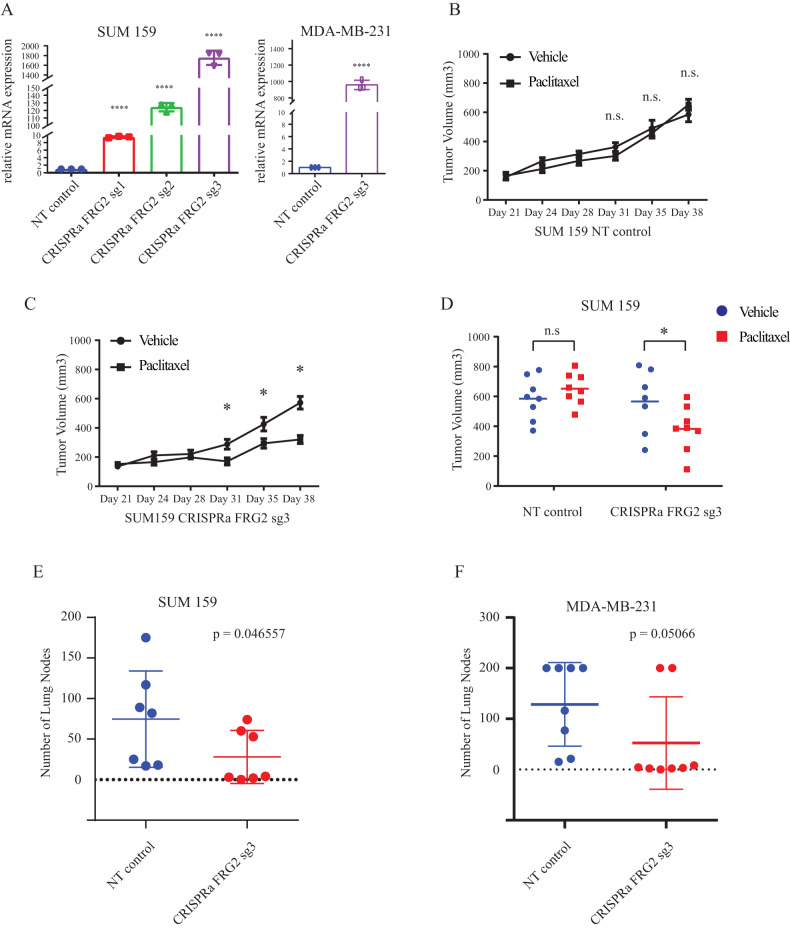
Endogenous activation of FRG2 gene expression sensitizes tumor to paclitaxel and inhibits metastasis. **A** FRG2 mRNA expression level of SUM159 (left) and MDA-MB-231 (right) quantified by RT-PCR. Data are presented as mean ± SD (*n* = 3). The *p* value is calculated by the two-sided Student’s *t* test. **B**–**D** CRISPRa FRG2 sg3 and NT control SUM159 cells were transplanted into NSG mice. Mice were split into vehicle and paclitaxel treatment group by averaging tumor volumes (7 or 8 mice for each group). Vehicle or paclitaxel (5 mg/kg) was intravenously injected twice per week. **B**, **C** Tumor volumes at different day points are represented as mean ± SEM. **D** The individual tumor volumes at experiment endpoint. The *p* values are calculated by the two-sided Student’s *t* test. **E**, **F** NT, CRISPRa FRG2 sg3 SUM159 and NT, CRISPRa FRG2 sg3 MDA-MB-231 cells were injected intravenously in NSG mice to assess lung metastatic nodule formation. Data are represented as individual dot plots and mean ± SD (*n* = 7 per group for SUM159, *n* = 8 per group for MDA-MB-231). The *p* value is calculated by the Mann–Whitney *U* test. n.s. *p* > 0.05, *p < 0.05, **p < 0.01, ***p < 0.001, or ****p < 0.0001.

The several candidate genes identified in our screens and study, FRG2 was the most potent at regulating paclitaxel response and metastasis (Fig. 4). As such, to further explore FRG2 therapeutic potential and gain further insights into its role and contribution toward paclitaxel resistance, we applied a complementary, alternative gain-of-function approach through endogenous activation of the FGR2 specific promoter, using the CRISPR/dCas9 Synergistic Activation Mediator (SAM) system, as shown previously [17]. Three distinct specific lentiSAM CRISPR sgRNAs targeting the FRG2 gene promoter were used in TNBC SUM159 cells (NT gRNA was used a negative control). As shown in Fig. 5A left panel, all 3 sgRNAs targeting the FRG2 gene promoter significantly increased FRG2 mRNA levels, compared to NT control. CRISPRa FRG2 sg3 showed the strongest increase in FRG2 mRNA expression and was further selected to be validated and confirmed in another TNBC cell line, MDA-MB-231 (Fig. 5A, right panel) as well as for all subsequent in vivo experiments in both cell lines. FRG2-activated (CRISPRa FRG2sg3) and NT SUM159 cells were orthotopically transplanted into NSG mice and animal were treated with a low dose of paclitaxel (5 mg/kg) or vehicle. As shown in Fig. 5B, at that low dosage, paclitaxel does not significantly reduce tumor size or volume in control (NT) animals. Interestingly, however, the paclitaxel response was significantly potentiated in the presence of increased FRG2 levels (Fig. 5C). Tumors were resected at end point showed a significant decrease in tumor volume distribution of the CRISPRa FRG2sg3 group treated with low dose of paclitaxel as compared to the NT control group (Fig. 5D).

Having shown that the FRG2 KO increased lung metastasis (Fig. 4), we next assessed whether FRG2 overexpression could prevent or inhibit metastatic lung colonization in preclinical models of lung metastasis. As shown in Fig. 5E, activation of the FRG2 endogenous promoter potently inhibited tumor metastasis and strongly reduced the numbers of lung metastatic nodules in SUM159 TNBC. These effects were extended to another model of TNBC lung metastasis, using the MDA-MB-231 cells (Fig. 5F). These results indicate that activation of the endogenous FRG2 gene promoter significantly decreased the numbers of lung metastatic nodules by 62% and 43% in SUM159 and MDA-MB-231 TNBC tumors, respectively.

Altogether, these results suggest that FRG2 could be potentially used as a prognostic marker to predict patients’ response to paclitaxel treatment and indicate that any means of increasing FGR2 endogenous expression levels could efficiently overcome paclitaxel resistance by sensitizing TNBC cells to drug treatment as well as limit the metastatic spread. As such, FRG2 can represent a valuable therapeutic target for the treatment of TNBC.

## Discussion

Taxane-based chemotherapy (i.e., paclitaxel) has been widely used in treatment for various types of cancer such as prostate, breast, lung cancer [46–48]. However, despite initial response, patients often start developing resistance to the drug, ultimately failing follow-up taxol treatments. The development of taxol drug insensitivity or resistance also increases potential risks of tumor relapse or distant metastasis, leading to poor clinical outcome [49, 50]. While several molecular mechanisms have been shown to contribute to chemoresistance (i.e., increased transporter pump activity, stemness, genetic alteration, altered DNA repair, epithelial-mesenchymal transition (EMT), and cancer stemness, the complete landscape contributing to paclitaxel resistance is not fully understood [51]. Thus, there is strong need for novel therapies targeting specific molecular features of TNBC to compensate for chemotherapy resistance for TNBC patients [52].

While RNA interference (RNAi) technology has proven useful in identifying chemotherapy regulators [53], it also has limitations as residual target expression may suffice to carry on biological functions [54]. Recently, CRISPR-based gene editing approaches gained lots of attention in forward genetic screens, due to their higher efficacy in knocking out specific genes, compared to more traditional RNAi knock-down approaches [55]. Recent large-scale genome-wide CRISPR screens performed in various types of solid tumors, including breast cancer, allowed for the identification of novel cancer vulnerabilities and the development of novel potential therapeutic treatment strategies for cancer patients [17, 56]. In this study we interrogated a genome-wide CRISPR library, under paclitaxel selection pressure, to identify potential drug sensitizer/resistance genes. CRISPR loss-of-function genetic screens were performed both in vitro and in vivo, allowing for the identification of specific genes involved in TNBC resistance and sensitivity to the paclitaxel treatment. Interestingly, we found several of our top targets to play a regulatory role in TNBC stemness. While breast tumors are heterogeneous in nature, they contain small subpopulations of stem-like breast cancer cells (BCSCs) that have been previously shown to be largely responsible for chemotherapy resistance [13]. Moreover, BCSC numbers are significantly increased in chemo-resistant cells or following chemotherapy treatment [34]. BCSCs exhibit tumor forming and self-renewal abilities as well as efficient DNA damage repair mechanisms, providing them with a survival advantage in cytotoxic environments [30]. Because BCSCs have high expression of adenosine triphosphate binding cassette (ABC) transporters, leading to high drug efflux they are also prone to evade apoptosis induced by chemotherapy drug treatments [57]. We found that ATP8B3, FOXR2, FRG2 and HIST1H1A gene silencing significantly enhanced TNBC cells tumor-initiating capacity as well as expression of the TNBC stemness marker, EPCR, thereby defining a new role for these genes in stemness regulation. Moreover, ATP8B3, FOXR2, FRG2 also decreased tumor response to paclitaxel in vivo, in preclinical models of TNBC tumorigenesis. Together, these results indicate that these newly identified stemness regulators act to prevent BCSC self-renewal activity and suggest that these genes could potentially enhance TNBC tumor response to paclitaxel and chemotherapy treatments. In future studies, it will be interesting to further analyze and investigate the molecular mechanisms leading to regulation of stemness downstream of each identified candidate genes through more detailed analysis of expression levels of well characterized stemness markers like SOX2, CD49f, CD44, OCT4, RAD18 and Nanog.

Chemo-resistant breast cancer cells can induce cancer stemness while enhanced cancer stemness potentiates chemoresistance [29, 34]. The reciprocal association between these two evolved features results in high risk of tumor propagation as demonstrated by BCSCs which are a leading cause of distant metastasis [58–60]. Stem related gene expression signatures have been found in metastatic cancer, and chemoresistance and metastasis are two tightly associated events during cancer development [61, 62]. We thus assessed whether our newly identified stemness regulatory genes could affect the metastatic process in TNBC. Using a preclinical, tail vein injection TNBC model of lung colonization, we found all 3 genes (ATP8B3, FOXR2, and FRG2) KOs to promote TNBC metastasis, suggesting these genes play a role as suppressors of metastasis in TNBC. This is particularly true for the FRG2 gene for which gene silencing resulted in the strongest prometastatic response. Given the strategy by which these genes were identified, through their ability to inhibit paclitaxel resistance, we suggest that the relationship between chemoresistance and suppression of metastasis can be further explored.

FRG2, facioscapulohumeral muscular dystrophy (FSHD) Region Gene 2, is a gene that was found transcriptionally activated in FSHD patients [63, 64]. Our results uncovered new functions for FRG2 in the context of breast cancer and chemotherapy. We found that FRG2 potently regulates breast cancer stemness, sensitizes breast tumors to chemotherapy treatments and prevents tumor formation and progression in TNBC. The proposed role of FRG2 as a potent suppressor of stemness is evidenced by the strong increase in cancer stem cell numbers and TBNC stemness marker expression when the FRG2 gene is silenced. Interestingly, the FRG2 gene was found to be induced in differentiated muscle cells [65]. Our results further suggest that FRG2 could act as a differentiation factor in breast cancer and prevent cancer stemness. Consistent with a role as a stemness suppressor, we further found that FRG2 also acts as a potent suppressor of tumor metastasis by efficiently preventing secondary lung metastatic nodule formation in preclinical models of TNBCs. Finally, we show that FRG2 can be used as a therapeutic target to overcome paclitaxel resistance and sensitize breast cancer cells to chemotherapy. Activating the endogenous FRG2 promoter to induce FRG2 gene expression significantly restored chemotherapy responses in resistant TNBC cells and led to a strong decrease in tumor volume following treatment with paclitaxel. Furthermore, and adding to the clinical relevance of our results, any therapeutic means of increasing FRG2 expression could enhance effectiveness of lower dosage, thus less toxic, of the chemotherapy drug paclitaxel in TNBC patients. Altogether, these results underscore the potential therapeutic value of FRG2 for chemotherapy treatments and prevention of metastasis in TNBC tumors.

## Materials and methods

### Cell lines and cell culture

Human breast cancer cell lines SUM159 were cultured in Ham’s F-12 nutrient mixture (WISENT INC.) containing 5% fetal bovine serum (FBS, Gibco), 5 µg/ml insulin, and 1 µg/ml hydrocortisone. Cell lines MDA-MB-231 and HEK293T were cultured in Dulbecco’s Modified Eagle’s Medium (DMEM, WISENT INC.) supplemented with 10% FBS. The SUM159 cell line was obtained from Stephen Ethier (The Medical University of South Carolina). Detailed information of the SUM159 cell line is available at Breast Cancer Cell Line Knowledge Base (www.sumlineknowledgebase.com). MDA-MB-231 was purchased from ATCC. HEK293T was obtained from Genhunter. All the cell lines were routinely tested by Diagnostic Laboratory from Comparative Medicine and Animal Resources Centre (McGill University).

### GeCKO v2 library cloning and library virus production

Human CRISPR Knockout Pooled Library A (GeCKO v2, #1000000048) was obtained from Addgene. Library A contains a total of 65,383 sgRNAs (3 sgRNAs for 19,050 genes, 4 sgRNAs for 1864 miRNAs and 1000 non-targeting control sgRNAs). The library virus was produced according to the published protocol. In brief, the library plasmids were electroporated into Stbl3 bacteria (Invitrogen), then transformed bacterial cells were plated on bioassay ampicillin plates for 14-h bacterial culture at 32 °C. The colonies were collected, and the plasmids were isolated and purified using Maxiprep kits (Qiagen). HEK293T cells were transfected with library plasmids, packaging vector psPAX2 and envelope vector pMD2.G. The virus-containing medium was harvested 48–72 h after transfection.

#### CRISPR library virus transduction and drug screen

In each independent experiment, we infected ~150 million SUM159 at MOI of 0.3–0.5; corresponding to a cell survival rate of 30–40%. Briefly, 3 million SUM159 cells were plated into each well of 12-well culture plates with 8 µg/ml of polybrene (EMD Millipore Corp. #TR-1003-G). The library virus was added based on the previously optimal titered concentrations allowing for a 30–40% cell survival rate. The plated cells were spin-infected at 1000 × *g* for 2 h at 32 °C and incubated at 37 °C overnight. Puromycin selection (2 µg/ml) was then performed for 7 days before the cells be divided into three groups. (1) 30 million transduced cells were collected for sequencing to assess library representation. (2) For in vitro drug screen, 40 million infected cells were cultured in T225 flasks in the presence of paclitaxel (10 nM) while another 40 million cells were cultured with vehicle (DMSO) treatment. Cell number counting was performed every 3 days for 2 weeks. (3) For each round of the in vivo screening, 30 million cells/mouse were transplanted subcutaneously into 4 mice. Once tumors were palpable, mice were treated with either paclitaxel (15 mg/kg) or vehicle. Paclitaxel and vehicle were administered once per week over 3 weeks. The mice were then sacrificed, and tumors were snap frozen at −80 °C for subsequent genomic DNA extraction and deep-sequencing.

#### Genomic DNA extraction from in vivo and in vitro samples

Genomic DNA was extracted using Qiagen Blood & Tissue Kit (Qiagen) and reference kit protocol was followed. Briefly, 6 ml of NK lysis buffer containing 50 mM Tris, 50 mM EDTA, 1% SDS @ pH 8 and 30 µl of 20 mg/ml Proteinase K (Qiagen) was used for the lysis of 30 million cells or 200 mg of grinded tumor samples. Cells were then incubated for 1 h at 55 °C. Tumors were incubated overnight at 55 °C. Cell lysates were incubated for another 30 min with RNAse A (Qiagen) at the final concentration of 0.05 mg/ml and then placed on ice for 10 min. After adding 2 ml of ice cold 7.5 M ammonium acetate (Sigma), the samples were vortexed and then centrifuged at 4000 × *g* for 10 min. The supernatants were collected and precipitated by mixing with isopropanol and then centrifuged at 4000 × *g* for 10 min. The pellets were kept and washed in 70% cold ethanol, air dried and resuspended in 500 µl 1 × TE Buffer at 65 °C for 1 h. The genomic DNA concentration was measured using Nanodrop (Thermo Fisher).

#### Library preparation for next generation sequencing

Two-step PCR was performed to prepare the samples for sequencing. The key principle for the first PCR reaction (PCR1) is that the input amount of genomic DNA for each sample must be sufficient to maintain the 300× coverage of the GECKO library. Each sample for sequencing was prepared in PCR1 reactions as follows: 98 °C for 2 min, 98 °C for 10 s, 60 °C for 20 s, 72 °C for 30 s, and 72 °C for 2 min for 18 cycles. Each 100 µl PCR1 reaction contained 20 µl Herculase 5× Buffer, 1 µl of 100 mM dNTP, 2.5 µl of Forward Primer F, 2.5 µl of Reverse Primer, 1 µl Herculase II Fusion Enzyme (Agilent), 10 µg of the extracted DNA and PCR grade water. The adapters specific to Illumina sequencing were attached in the second PCR (PCR2). Each 100 µl PCR2 reaction (20 µl Herculase 5× Buffer, 1 µl of 100 mM dNTP, 2.5 µl of Forward Primer, 2.5 µl of Reverse Primer, 1 µl Herculase II Fusion Enzyme, 5 µl of PCR1 amplicon and 68 µl of PCR grade water) was performed in the same way as PCR1 reaction. The resulting PCR products were run on a 2% agarose gel, then gel extracted and purified using PCR & Gel Cleanup Kit (Qiagen). The library-ready samples were sequenced at Génome Québec (https://www.genomequebec.com/) and 20 million reads capacity was assigned to each sample.

#### Individual CRISPR knockout and activation plasmid cloning and lentivirus production

For knockout lentivirus, LentiCRISPR v2 backbone vector was obtained from Addgene (plasmid # 52961). For activation lentivirus, LentiSAM v2 (plasmid #75112) and LentiMPH v2 (plasmid # 89308) were obtained from Addgene. Both knockout and activation sgRNA plasmid cloning procedures followed the Golden Gate cloning protocol [66]. Briefly, the pair of oligo primers for each gene was phosphorylated and annealed in presence of T4 PNK enzyme. Reactions were then incubated at 37 °C for 30 min, 95 °C for 5 min and ramped down to 25 °C at 5 °C/min on a thermal cycler (Bio-Rad). The annealed oligos were diluted 1:10. Golden Gate assembly reaction was performed on the thermal cycler (Bio-Rad). Each reaction contained T7 ligase (Enzymatics), Restriction enzyme (NEB), BSA (NEB), rapid ligase buffer (Enzymatics), annealed oligos, and backbone vector. Each cycle was run at 37 °C for 5 min and 20 °C for 5 min and repeated for a total of 15 cycles. The cloned vectors were further transformed into Stbl3 bacteria (Invitrogen) and seeded on LB agar plates with ampicillin at 33 °C overnight. HEK293T cells were transfected with cloned vector, pMD2.G (Addgene #12259) and psPAX2 (Addgene #12260). After overnight incubation, the culture medium was changed with fresh medium. The supernatant was collected from the culture plates after another 24-h incubation.

**Table:** 

Knockout primers	Sequence
ARHGEF39_F_KO	caccgCCGGAGGTTTGTACGGCTTC
ARHGEF39_R_KO	aaacGAAGCCGTACAAACCTCCGGc
ATP8B3_F_KO	caccgTCCTCTTCATCCGTGCCACC
ATP8B3_R_KO	aaacGGTGGCACGGATGAAGAGGAc
DHRS7_F_KO	caccgAACCAGTGTCGGTCAGGTCA
DHRS7_R_KO	aaacTGACCTGACCGACACTGGTTc
DIO3_F_KO	caccgCACATCCTCGACTACGCGCA
DIO3_R_KO	aaacTGCGCGTAGTCGAGGATGTGc
FOXR2_F_KO	caccgCACGAGTCTCCTCCCAAAAG
FOXR2_R_KO	aaacCTTTTGGGAGGAGACTCGTGc
FRG2_F_KO	caccgACAGATCTCCTTTACAGAAA
FRG2_R_KO	aaacTTTCTGTAAAGGAGATCTGTc
HIST1H4A_F_KO	caccgGATCTCTGGTCTGATCTACG
HIST1H4A_R_KO	aaacCGTAGATCAGACCAGAGATCc
HRG_F_KO	caccgCATCAGCAATCCGCAGCAAT
HRG_R_KO	aaacATTGCTGCGGATTGCTGATGc
HSPA13_F_KO	caccgGATGACCATCGCGTGAACAG
HSPA13_R_KO	aaacCTGTTCACGCGATGGTCATCc
IFNE_F_KO	caccgCCAGTCCCATGAGTGCTTCT
IFNE_R_KO	aaacAGAAGCACTCATGGGACTGGc
ITGB6_F_KO	caccgGGCATCGTCATTCCTAATGA
ITGB6_R_KO	aaacTCATTAGGAATGACGATGCCc
NDUFC2_F_KO	caccgTCGCCAGCTTCTATATATTA
NDUFC2_R_KO	aaacTAATATATAGAAGCTGGCGAc
NOTCH2_F_KO	caccgTTGATGACTGCCCTAACCAC
NOTCH2_R_KO	aaacGTGGTTAGGGCAGTCATCAAc
PDLIM2_F_KO	caccgAGTGCTGGCGACTCGCTTCC
PDLIM2_R_KO	aaacGGAAGCGAGTCGCCAGCACTc
PHACTR1_F_KO	caccgGGCGTCACCTTCCGTTGCTA
PHACTR1_R_KO	aaacTAGCAACGGAAGGTGACGCCc
RGN_F_KO	caccgCCCGCCGGGAGGTACTTTGC
RGN_R_KO	aaacGCAAAGTACCTCCCGGCGGGc
SLC36A3_F_KO	caccgCAACAAGCCGGCATTCTTTA
SLC36A3_R_KO	aaacTAAAGAATGCCGGCTTGTTGc
SOGA2_F_KO	caccgCCTCCACCGTCTTAAGTTCG
SOGA2_R_KO	aaacCGAACTTAAGACGGTGGAGGc

**Table:** 

Activation primers	Sequence
FRG2a_sg1_F	caccgGAGCACAGGGACCGGAAAAT
FRG2a_sg1_R	aaacATTTTCCGGTCCCTGTGCTCc
FRG2a_sg2_F	caccgGCACAGGGACCGGAAAATCG
FRG2a_sg2_R	aaacCGATTTTCCGGTCCCTGTGCc
FRG2a_sg3_F	caccgTTGAGGCTCTAAGAAGCGGC
FRG2a_sg3_R	aaacGCCGCTTCTTAGAGCCTCAAc

#### In vivo Xenograft studies and drug treatments

All animals were housed and handled in accordance with the approved guidelines of the Canadian Council on Animal Care (CCAC) “Guide to the Care and Use of Experimental Animals”. All experiments were performed under the approved McGill University Animal Care protocol (AUP # 7497 to JJL). All transplantation procedures were undertaken using isoflurane anesthesia. SUM159 cells infected with GECKO library were prepared in PBS (Phosphate Buffered Saline 1X, WISENT INC.) and transplanted into NSG mice by means of subcutaneous injection. For single KO cell transplantation, 1 million SUM159 cells were initially diluted in 20 µl PBS and 20 µl Matrigel (BD Bioscience) and then transplanted into mammary glands of NSG mice. When the tumors became palpable, after 3–4 weeks, paclitaxel (Sigma) and vehicle (control) were intraperitoneally administered twice per week. Paclitaxel was dissolved in 10% DMSO (Sigma), 40% PEG300 (Sigma), 5% Tween-80 (Sigma) and 45% saline. The mice were treated for 2–3 weeks before tumors reached maximum volume of 1000 mm^3^ and then were euthanized. Tumor volumes were documented. For tail vein injection, 1 million cells were prepared in 100 µl PBS and injected into the median tail vein. The mice were euthanized after ~4 weeks and the lung tissues were collected and stained in Bouin’s solution (Sigma) for at least 48 h. Lung metastatic nodules were counted under a microscope.

#### Cell viability assay

Infected SUM159 or MDA-MB-231 cells were seeded into 96-well plates at the density of 5000 cells per well. Cells were treated with DMSO (control) or Paclitaxel (10 nM) after 24 h cell attachment. After 72 h treatment, 7% PrestoBlue Cell Viability Reagent (Invitrogen) was prepared in complete medium and 100 µl of the prepared reagent was added to each well. The cells were incubated at 37 °C for 1 h. Fluorescence was measured using the microplate reader (Tecan) at 535 nm excitation and 615 nm emission.

#### Tumorsphere assay

SUM159 cells were seeded into the ultra-low-attachment 24-well plate at the density of 10,000 cells/well. The culture medium contains HAM’s F12 medium (WISENT INC.), 10 ng/ml EGF (Invitrogen), 10 ng/ml bFGF (Invitrogen) and 1 × B27 (Invitrogen). After 7 days of culture, the number of tumorspheres were counted. Sphere-forming efficiency was calculated as: SFE (%) = number of spheres / number of cells plated × 100%.

#### Flow cytometry

Monolayer cells were dissociated into single cells and filtered through a 40 µm cell strainer. In total, 500,000 cells were incubated in prechilled PBS with 2% FBS for half an hour at 4 °C. Cell samples were further incubated with anti-EPCR for 30 min. The non-stained or single-stained samples were used as negative controls. Cells were then washed 3 times with FACS buffer and analyzed with BD FAC SCanto II cytometer (BD Biosciences) and Flowjo software (Tree Star Inc.).

#### Real-time PCR

Cells were lysed by TRIzol reagent (Invitrogen), and the total RNA was extracted following the standard procedures. In brief, Reverse Transcription (RT) was performed in each reaction containing RT buffer, 0.1 M DTT, Random hexamers, dNTP, ultrapure water (GIBCO) and M-MLV Reversed Transcriptase (Invitrogen). The real-time PCR was performed with SsoFastTM EvaGreen® Supermix (Bio-Rad) using a RotorGene 6000 PCR thermocycler. The RT-PCR steps are: 95 °C for 30 s, 40 cycles of 95 °C for 5 s, and 60 °C for 20 s. The paired primers are listed as follows.

**Table:** 

FRG2_forward	AAAGGCAAGCAGGATCGGAG
FRG2_reversed	AGCCCTGGAATGTCCCCTAT

#### Genomic cleavage detection (SURVEYOR) assay

GeneArt® Genomic Cleavage Detection Kit (Invitrogen) was used for SURVEYOR assay and detailed procedures were according to the manufacture’s protocol. In brief, cell samples were lysated using cell lysis buffer (2 µl protein grader in 50 µl cell lysis buffer). PRC program (68 °C for 15 min, 95 °C for 10 min and 4 °C for holding) was run for DNA extraction. The PCR amplification was run on the program (95 °C for 10 min, the cycle of 95 °C for 30 s, 55 °C for 30 s and 72 °C for 30 was repeated 40 times and final extension 72 °C for 7 min). The PCR products was combined with detection reaction buffer. The re-annealing reaction was run on the PCR program (95 °C for 5 min, 95–85 °C at decreasing 2 °C/s, 85–25 °C at decreasing 0.1 °C/s and final holding at 4 °C). Detection enzyme was added to all test samples while water for all the negative control samples. After incubation at 37 °C for 1 h, the products were loaded and run in DNA electrophoresis gel for ~30 min at low voltage. The image was taken by imaging system (Bio-Rad).

#### Data processing and bioinformatic analysis

The bioinformatic tool, Cutadapt (https://cutadapt.readthedocs.io/en/stable/index.html), was initially used to demultiplex raw FASTQ files. Processed FASTQ files containing only the 20-nucleotide sgRNA sequence were then aligned to the library using MAGeCK count command. MAGeCK robust rank aggregation (RRA) was adopted to analyze abundance change of the sgRNAs and genes.

#### Correlational analysis of mRNA and paclitaxel response

PRISM drug response and mRNA data were downloaded from DepMap portal (https://depmap.org/portal/). Paclitaxel drug response (EC50) and mRNA profiles of the target genes were extracted from the breast cancer datasets. Integrating EC50 and mRNA data results a file containing 34 genes’ mRNA across 42 breast cancer cells and paclitaxel EC50 (Supplementary File 4). For each gene, correlation was calculated between mRNA and paclitaxel EC50 across breast cancer cell lines.

#### Statistical analyses

Statistical analysis was done with GraphPad Prism software 9.0. 0. All in vitro experiments were done at least three independent times. For animal studies, at least five independent animals were used in each condition group. The sample sizes were chosen empirically based on the previous observations. No randomization was needed in the study. If not stated otherwise, all data presented mean ± SD. Each data point represents an individual animal or an independent experiment. If not stated otherwise, the unpaired *t-*test (two-tailed) was applied to compare the means of two groups. *p* values < 0.05 were considered significant (*p < 0.05; **p < 0.01; ***p < 0.001; ****p < 0.0001).

### Supplementary information

Suppl. file 1

Suppl. file 2

Suppl. file 3

Suppl. file 4

Suppl. doc

## Data Availability

The data used and/or analyzed during the current study are available and included in the Supplementary Information.
