# Supplementary material for: Combined in vitro/in vivo genome-wide CRISPR screens in triple negative breast cancer identify cancer stemness regulators in paclitaxel resistance
Source: Oncogenesis. 2023 Nov 6;12(1):51. doi: 10.1038/s41389-023-00497-9 (PMC10628277; doi:10.1038/s41389-023-00497-9)
Supplement: Supplementary file 5 — Suppl. doc [file 41389_2023_497_MOESM5_ESM.docx]

**Supplementary file 1**

sgRNA list of negative selection in cell (*in vitro*). Data was analyzed by MAGeCK package. The resulting sgRNA list was selected by three criteria as described. The final list contains 5288 sgRNA candidates.

**Supplementary file 2**

sgRNA list of positive selection in cell (*in vitro*). Data was analyzed by MAGeCK package. The resulting sgRNA list was selected by three criteria as described. The final list contains 10750 sgRNA candidates.

**Supplementary file 3**

sgRNA list of positive selection in tumor (*in vivo*). Data was analyzed by MAGeCK package. The resulting sgRNA list was selected by three criteria as described. The final list contains 141 sgRNA candidates.

**Supplementary file 4**

Data file containing 34 genes’ mRNA across 42 breast cancer cells and paclitaxel EC50 after Integrating EC50 of PRISM and mRNA data of CCLE.
